# Conservation/Mutation in the Splice Sites of Cytokine Receptor Genes of Mouse and Human

**DOI:** 10.1155/2013/818954

**Published:** 2013-12-17

**Authors:** Rosa Calvello, Antonia Cianciulli, Maria Antonietta Panaro

**Affiliations:** Department of Biosciences, Biotechnologies and Biopharmaceutics, University of Bari, Via Orabona 4, 70126 Bari, Italy

## Abstract

Conservation/mutation in the intronic initial and terminal hexanucleotides was studied in 26 orthologous cytokine receptor genes of Mouse and Human. Introns began and ended with the canonical dinucleotides GT and AG, respectively. Identical configurations were found in 57% of the 5′ hexanucleotides and 28% of the 3′ hexanucleotides. The actual conservation percentages of the individual variable nucleotides at each position in the hexanucleotides were determined, and the theoretical rates of conservation of groups of three nucleotides were calculated under the hypothesis of a mutual evolutionary independence of the neighboring nucleotides (random association). Analysis of the actual conservation of groups of variable nucleotides showed that, at 5′, GTGAGx was significantly more expressed and GTAAGx was significantly less expressed, as compared to the random association. At 3′, TTTxAG and xTGCAG were overexpressed as compared to a random association. Study of Mouse and Human transcript variants involving the splice sites showed that most variants were not inherited from the common ancestor but emerged during the process of speciation. In some variants the silencing of a terminal hexanucleotide determined skipping of the downstream exon; in other variants the constitutive splicing hexanucleotide was replaced by another potential, in-frame, splicing hexanucleotide, leading to alterations of exon lengths.

## 1. Introduction

In most protein-coding genes of eukaryotes the coding exons alternate with noncoding introns. The nuclear pre-mRNA is a transcript of the whole gene, including the introns. However, before it is exported to the cytoplasm, the introns are removed through a process called pre-mRNA splicing and the exons orderly joined to form the mature coding mRNA. Cutting at splicing sites is usually accomplished with a high degree of precision, as needed for the synthesis of the correct protein products in the process of translation. Precision splicing requires the existence of specific sequence arrangements at appropriate pre-mRNA sites (signals) and is affected by a massive ribonucleoprotein complex, the spliceosome, which has evolved to interact with these sequences. Most often the splicing signal is univocal and robust enough to allow one single splicing pattern only at each site. But when the splicing signals are less robust or possibly not univocal, “physiological” alternative splicing patterns may occur, with total or partial deletion of some exons or retention of in-frame introns resulting in alterations of the encoded protein product. In most of these cases the generated protein either retains a similar function to that of the default protein or may acquire a different biological function [1–3]. In other instances “unsuited” mRNAs are prevented from crossing the nuclear membrane, a selection structure which emerged in eukaryotes to separate intron-containing RNAs from the translation apparatus. In addition, other cellular systems may degrade irregularly spliced or mutated mRNAs (nonsense-mediated decay, NMD) [4–6] or, eventually, altered proteins may be ubiquitinated and proteasome-degraded [7]. However, in some cases, despite all these control mechanisms, an irregular splicing or a disruption of the physiological alternative splicing may impair cell functions and bring about severe illnesses [8–10]. Special strategies to allow some unspliced virus-derived mRNAs (required for the synthesis of some envelope and capsid proteins) to be exported out of the nucleus are adopted by human immunodeficiency virus type 1 [11].

The main intronic splicing signals are the 5′ splice site (5′ss) or splice donor site; the branch site; the polypyrimidine tract; and the 3′ splice site (3′ss) or splice acceptor site. The 5′ss marks the 5′ end (beginning) of the intron and in almost 99% of cases begins with the “canonical” dinucleotide GT, followed by a few varying nucleotides. The branch site is a very short sequence including an adenine nucleotide. The polypyrimidine tract is a ~15-nucleotide sequence with a rich content of Cs and Ts. The 3′ss marks the 3′ end of the intron and in almost 99% of cases it ends with the “canonical” dinucleotide AG, which is preceded by a few varying nucleotides. The polypyrimidine tract is located immediately upstream of the 3′ss and the branch site lies further upstream at a short distance. In 1% of the introns the canonical 5′ss-3′ss combination GT–AG is replaced by noncanonical combinations, such as GC–AG or AT–AC.

Besides the above-mentioned main splicing signals, it is believed that other sections of the intron may harbor additional “splicing motifs” which may contain consensus sequences for specific nuclear proteins. In addition, both introns (I) and exons (E) possibly harbor splicing enhancer (ISE and ESE) and splicing silencer (ISS and ESS) motifs. However, these splicing motifs are neither specific nor constantly present and the “splicing code” may be context-dependent and result from an integration of several different inputs [12–23].

The spliceosome is a massive ribonucleoprotein complex comprising some small “U”s nuclear RNAs (snRNAs) plus different specific proteins, these complexes being referred to as small nuclear ribonucleoproteins (snRNPs or SNURPs). In addition, more than a hundred other protein factors cooperate in splicing. Two types of spliceosomal introns have been described: U2 snRNP-dependent introns (the main group) which use U1, U2, U4, U5 and U6 snRNPs (with three subtypes, according to the terminal dinucleotides: GT–AG, GC–AG and AT–AC) and U12 snRNP-dependent introns which use U11, U12, U4atac, U6atac and U5, snRNPs (with two subtypes, according to the terminal dinucleotides: AT–AC and GT–AG) [12–15, 24–27].

Large-scale statistical analyses of the splice sites have been made in model species of vertebrates, invertebrates, fungi, protozoa, and plants [25–30]. Comprehensive databases have also been generated, for example, http://www.softberry.com/spldb/SpliceDB.html [29–31], and [26]. Cumulatively, the above-quoted papers report data on the global nucleotide patterns at the splice sites in each species, that is, the frequency of each base at a given position with reference to the intron boundaries (including the flanking sections of the neighboring exons), the information content of the intronic sequences which are bound by the splicing factors (as a measure of their conservation), and the evolution of the splicing factors in parallel with the evolution of the intronic splicing signals. We deemed that a comparative analysis of the intronic splice signals in orthologous genes (i.e., genes which derived from a common ancestor and diverged following speciation events) at the individual orthologous splice sites could throw further light on the evolution of the splice sites during the process of speciation. In some topographically corresponding splice sites of orthologous genes of Mouse and Human, the signaling sequences are identical, whereas in others they are different. It is likely that, at least in the great majority of cases, the unaltered sequences are derived from the common ancestor and were conserved during the process of divergent speciation. Thus, comparative analysis of orthologous splice sites could indicate which specific signaling sequences tend to be more conserved and thence the direction of the selective pressure in the time interval from the beginning of the speciation process to the present day. To this end we considered a group of cytokine receptor genes which are orthologous in Mouse and Human, two species which diverged quite recently, some 65–85 MYA (million years ago) [32, 33]. We also examined the Mouse and Human transcript variants in this group of receptors with the aim of spotting those splicing signals which are more frequently silenced or replaced by other potential splicing signals at a different position in the gene.

## 2. Materials and Methods

This study was made on a group of orthologous genes of Mouse and Human coding for cytokine receptors [34, 35]. (A database of homologous genes is available at http://www.ncbi.nlm.nih.gov/homologene/; a database of orthologous genes is available at http://inparanoid.sbc.su.se/cgi-bin/index.cgi/.) A structural classification of cytokine receptors (mainly according to Coico and Sunshine 2009) divides the receptors into the following groups: (1) immunoglobulin superfamily receptors; (2) class I cytokine receptor family; (3) class II cytokine receptor family (Interferon receptor family); (4) tumor necrosis factor receptor superfamily; (5) chemokine receptor family; (6) transforming growth factor beta receptor family. In this study we analyzed 26 receptors, belonging to all these groups, as indicated in Table 1. Sequences were retrieved from the NCBI GenBank (http://www.ncbi.nlm.nih.gov/). We selected the splice variants of these genes which showed superimposable exon-intron arrangements in Mouse and Human and exons aligning with nucleotide identities of 70% or higher. Eventually, 216 Mouse/Human couples of introns were extracted. In addition, we compared these canonical transcripts with the Mouse and Human transcript variants of the same genes.

Nucleotide sequence alignments without gaps of each couple of orthologous introns were made using a program available at http://multalin.toulouse.inra.fr/multalin/ [36].

All probabilistic comparisons between percentages were made using the binomial distribution (one-tailed tests) with the original actual figures [37].

## 3. Results

### 3.1. Nucleotide Identities at Corresponding Positions at the Ends of Mouse and Human Introns

**Figure 1:**
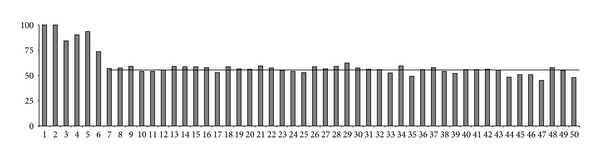
Nucleotide identities (%) in the first 50 nucleotides of each Mouse/Human couple of orthologous introns, indicating the probability of nucleotide conservation at any given position.

Nucleotide identities were recorded in orderly manner in the first 50 nucleotides of each Mouse/Human couple of introns (Figure 1). All the 216 couples of introns considered started with the canonical dinucleotide GT and identity was, of course, 100% at positions 1 and 2. Then identities averaged 84.3%, 90.3%, 93.5%, and 73.6% at positions 3, 4, 5, and 6, respectively. In positions 7 to 50, the average identities were lower and remained relatively constant with a mean value of 55.4% (the horizontal line in Figure 1).

**Figure 2:**
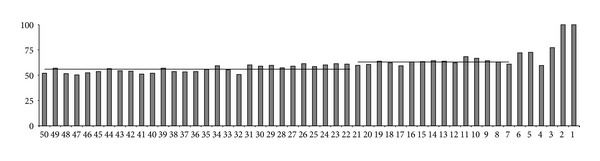
Nucleotide identities (%) in the last 50 nucleotides of each Mouse/Human couple of orthologous introns, indicating the probability of nucleotide conservation at any given position.

Nucleotide identities were also recorded in the last 50 nucleotides (numbered from 50 to 1) of each Mouse/Human couple of introns (Figure 2). All couples of introns ended with the canonical dinucleotide AG and thus identity was 100% at positions 1 and 2. Then identities averaged 77.3%, 59.7%, 72.7%, and 72.2% at positions 3, 4, 5, and 6, respectively. In positions 7 to 21 the average identities remained relatively constant with a mean value of 63.1% (the right horizontal line in Figure 2). In the last positions (22 to 50) the average identities remained relatively constant with a mean value of 56.0% (the left horizontal line in Figure 2).

From these preliminary data as well as from other literature data [25] the initial and terminal hexanucleotides of the introns appeared to be the best characterized components of the 5′ss and the 3′ss, respectively. We thus undertook a more detailed analysis of these sequences.

### 3.2. The Initial Hexanucleotides of the Introns

The possible number of hexanucleotides beginning with GT is 4^4^ = 256. Of these, 51 (cumulatively in Mouse and Human) were found in our sample, which does not exclude the possibility that other hexanucleotides are used in other genes. The more frequent initial hexanucleotides are listed in Table 2. The per cent incidences of these hexanucleotides in Mouse and Human do not differ significantly (*P* > 0.05).

The nucleotide composition according to the position in the initial hexanucleotides in Mouse and Human is shown in Table 3, columns 1–4. The per cent incidences of the different nucleotides in Mouse and Human at each position did not differ significantly (*P* > 0.05) except in the case of the sixth nucleotide, where C was significantly more represented in Mouse as compared to Human.

### 3.3. The Terminal Hexanucleotide of the Introns

Of the 256 possible hexanucleotides ending with AG, 94 (cumulatively in Mouse and Human) were found in our sample, which does not exclude the possibility that other hexanucleotides are used in other genes. The more represented terminal hexanucleotides are listed in Table 4. The per cent incidences of these hexanucleotides in Mouse and Human did not differ significantly (*P* > 0.05).

The nucleotide composition according to the position in the terminal hexanucleotides in Mouse and Human is shown in Table 5, columns 1–4. The per cent incidences of the different nucleotides in Mouse and Human at each position did not differ significantly (*P* > 0.05) except in the case of the first nucleotide, where G was significantly more represented in Mouse as compared to Human. It is noteworthy that in our sample the nucleotide G was never present in the fourth position so that no hexanucleotide ended by GAG.

The limits of the polypyrimidine tract are not well defined and the two leftmost nucleotides (C- and T-rich positions 1 and 2; Table 4) of the terminal hexanucleotide might, in fact, be the rightmost part of the polypyrimidine tract.

### 3.4. The “Bifunctional” (Initial or Terminal) Hexanucleotides of the Introns

All the 16 (4^2^) hexanucleotides beginning by GT and ending by AG may in theory act as both 5′ss and 3′ss (“bifunctional” hexanucleotides). Of these, 8 never appeared as initial or terminal hexanucleotides in our sample and 3 (GTAAAG, GTACAG, and GTTAAG) appeared at the beginning (but never at the end) of some introns, while 5 (GTCCAG, GTGCAG, GTGTAG, GTTCAG, and GTTTAG) appeared at the end (but never at the beginning) of other introns, in both Mouse and Human.

### 3.5. Conservation of the Individual Nucleotides in the Initial Hexanucleotides of the Introns

**Figure 3:**
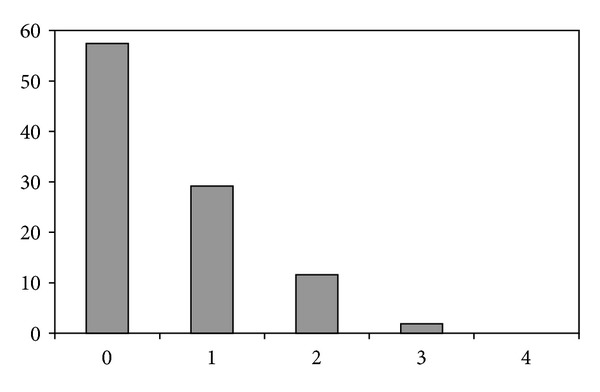
Abscissa: number of nucleotide differences (irrespective of the position) in orthologous Mouse/Human initial hexanucleotides. Ordinate: percentage.

The structural conservation/mutation in the couples of Mouse/Human orthologous initial hexanucleotides was studied. In more than half of the couples (57.4%) there was a complete identity between the Mouse and Human orthologous hexanucleotides. A decreasing percentage of couples exhibited one, two, or three nucleotide differences (regardless of position); in no instance all four variable nucleotides changed (Figure 3). The number of changes per hexanucleotide averaged 0.58.

We calculated the probability of random occurrence of the same nucleotide at a given position in orthologous Mouse/Human hexanucleotides (Table 3, column 5). For instance, A in the third position is present with probability 0.597 in the Mouse and 0.570 in the Human (Table 3, GTAxxx, columns 2 and 3); then the probability of random occurrence of A at that position in both Mouse and Human is 0.597 × 0.570 = 0.3403 (or 34.03%) (binomial distribution; *n* = 216). The actual occurrence (%) of nucleotide identities at each position is reported in column 6 of Table 3. The percentages of actual identities were always significantly (*P* < 0.01) higher than the percentages of random identities and the difference expresses the level of nucleotide biological conservation. In the nucleotides of the initial hexanucleotides the actual total conservation was about 53% higher than the calculated purely random conservation.

### 3.6. Conservation of Groups of Nucleotides in the Initial Hexanucleotides of the Introns

The contribution of groups of bases in the initial hexanucleotides to enhancing or reducing the level of Mouse/Human conservation was then studied. The random probability of occurrence of complete hexanucleotides or groups of three or two nucleotides at given positions was calculated on the basis of the percentages of the actual conservation of the individual component nucleotides at the corresponding positions, as reported in Table 3 (column 6). For example, the random probability of the hexanucleotide GTAAGT is

(1) 1.0000×1.0000×0.5093×0.7778   ×0.8241×0.4537=0.1481  (or  14.81%)

(binomial distribution; *n* = 216) and the random probability of the sequence GTAAGx is

(2) 1.0000×1.0000×0.5093×0.7778×0.8241 =0.3265  (or  32.65%).

The expected random probabilities were compared with the actual frequencies. Analysis of the initial hexanucleotides showed that the sequences GTGAGx, GTGAxT, and GTGxGT are significantly more expressed than could be expected (Table 6). Analysis of all the hexanucleotides which included these sequences demonstrated that in GTGAGT (which included all three overexpressed sequences) the overexpression was highly significant (expected 8.75%; actual: 13.89%; *P* < 0.01); overexpression of GTGAGG (which included the GAG motif only) was less significant, possibly due to the lower frequency (expected 1.61%; actual 3.24%; *P* = 0.06). None of the other hexanucleotides which included some of the overexpressed sequences exhibited significantly different expression levels from those expected, due to the fact that they contained other not overexpressed or underexpressed motifs.

Sequences GTAAGx and GTAxGG were found to be significantly less expressed than could be expected (Table 6). The hexanucleotide GTAAGG, which included both underexpressed sequences, was clearly underexpressed, although at a low significance level due to the low frequency (expected 2.72%; actual 0.93%; *P* = 0.07). All other hexanucleotides which included some of the underexpressed sequences did not exhibit significantly different expression levels from those expected.

Another observation supports the role of the trinucleotides GAG and AAG in the association to overexpression and underexpression, respectively. The hexanucleotides GTGAGT and GTAAGT differ at the third nucleotide only. As at the third nucleotide the actual conservation of A is 1.69 times the actual conservation of G (50.93/30.09; Table 3), GTAAGT could be expected to be more expressed than GTGAGT by about 70%. On the contrary, GTGAGT was actually more expressed than GTAAGT by about 2% (18.5/18.1; Table 2) and indeed it was the most frequently expressed initial hexanucleotide. Similarly, GTAAGG should be more expressed than GTGAGG by 70%, but actually GTGAGG was more expressed than GTAAGG by about 27% (5.6/4.4; Table 2).

### 3.7. Conservation of the Individual Nucleotides in the Terminal Hexanucleotides of the Introns

**Figure 4:**
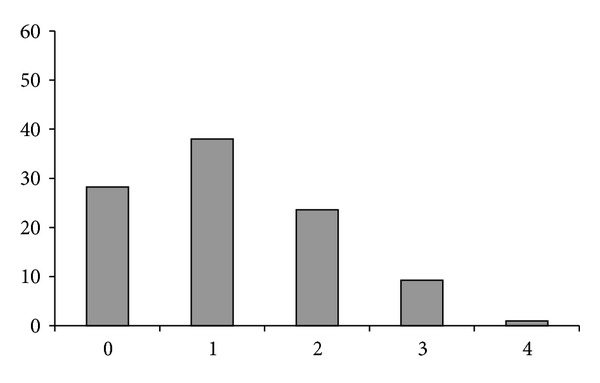
Abscissa: number of nucleotide differences (irrespective of the position) in orthologous Mouse/Human terminal hexanucleotides. Ordinate: percentage.

Conservation/mutation in the couples of Mouse/Human orthologous terminal hexanucleotides was also studied. In 28.2% only of the couples was there a complete identity between the Mouse and Human orthologous hexanucleotides. In a higher percentage of cases (37.9%) one nucleotide (regardless of position) was changed. A decreasing percentage of couples exhibited two or three nucleotide differences and in only 0.9% of cases were all four variable nucleotides changed (Figure 4). The number of changes per hexanucleotide averaged 1.17.

The probability of random occurrence of the same nucleotide at a given position in orthologous Mouse/Human hexanucleotides was calculated as previously reported (Table 5, column 5). The actual occurrence (%) of nucleotide identities at each position is reported in column 6 of Table 5. The percentages of actual identities were always significantly (*P* < 0.01) higher than the percentages of random identities. In the nucleotides of the terminal hexanucleotides the actual total conservation was about 80% higher than the calculated purely random conservation.

### 3.8. Conservation of Groups of Nucleotides in the Terminal Hexanucleotides of the Introns

The contribution of groups of bases in the terminal hexanucleotides in favoring the Mouse/Human conservation was studied by comparing their expected random probabilities with the observed actual frequencies (see previous paragraph for the computing of random probabilities and Table 5, column 6, for the actual conservation of the individual nucleotides at the different positions).

Analysis of the terminal hexanucleotides showed that the nucleotide sequences TTTxAG, TTxTAG, xTTTAG, TxTTAG, and xTGCAG are significantly more expressed than could be expected (Table 7). Furthermore, some dinucleotides derived from the above sequences are themselves overexpressed: TTxxAG (derived from TTTxAG and TTxTAG), xxTTAG (derived from xTTTAG and TxTTAG), and xTGxAG (derived from xTGCAG) (Table 7).

Three hexanucleotides were found to be significantly overexpressed as compared to the random probability: TTTCAG (expected 1.74%; actual 4.63%; *P* < 0.01); TTTTAG (expected 0.72%; actual 3.70%; *P* < 0.01); and CTGCAG (expected 0.54%; actual 1.85%; *P* = 0.03). All these hexanucleotides contained at least one of the conserved motifs. Other hexanucleotides, although containing some of the overexpressed trinucleotides, did not exhibit significantly different conservation levels from those of the random probability, and in no case did the occurrence of one of the conserved dinucleotides determine a higher conservation of the corresponding hexanucleotides.

No motif associated with a significant underexpression was found.

### 3.9. Splicing Variants

Splicing variants of the individual genes are usually of a different type in Mouse and Human so the positions of the splicing sites no longer correspond in the two species (i.e., the orthology of the splicing sites is lost), even though the main traits of the protein products are preserved. In only one instance, in receptor KIT, Mouse and Human exhibited the same type of variant, that is, the use of another 5′ss 12 nucleotides upstream of the canonical site.

In several cases one entire exon present in the canonical form is lacking in a variant although the other downstream exons are regularly transcribed, indicating that the constitutive 3′ss at the end of the preceding intron did not operate. As an example, in the transcript variant X1 (XM_005252446) of the Human IL2RA the canonical 3′ss TTCCAG immediately upstream of exon 4 is silenced and the fourth exon (216 nt; 72 aa) of the canonical sequence (NM_000417) is lacking in this variant. In other cases up to three consecutive 3′sss are silenced: in the Human IL2RG transcript variant X2 (XM_005262262) 3′sss ATCTAG, CTCTAG, and CTCCAG are all silenced, with loss of exons 2, 3, and 4, while exons 5, 6, 7, and 8 are transcribed as in the canonical form (NM_000206) without alteration of the reading frame.

In other cases the active alternative ss may be located upstream or downstream of the canonical ss, resulting in an alteration of the length of individual exons, usually without frameshifts. For example, in the Human variant X1 of CSF2RB (XM_005261340) the intron upstream of exon 7 ends by an “early” CCTCAG while the corresponding intron in the canonical sequence (NM_007780) ends at another CCTCAG 18 nt downstream, resulting in an 18-nt longer exon in the variant. Although variations of the 3′sss are more frequent, the 5′sss may also be variable. As an example, in Human transcript variant X1 of CD40 (XM_005260617) the 5′ss after exon 7 is GTGGGG, replacing the GTGAGT of the canonical variant (NM_001250) which occurs 12 nt upstream.

Detailed results are shown in the Supplementary Material available at http://dx.doi.org/10.1155/2013/818954.

### 3.10. The Binding of the U1 snRNA

We studied the ungapped base-pairing between the U1 snRNA sequence ACTTAC (NCBI GenBank accession code NR_004430), conserved in Mouse and Human, and the last three nucleotides of the exons plus the corresponding 5′ss hexanucleotide. The 5′ss GTAAGT, which exactly base-pairs with the U1, is expressed in 19.0% of cases in Mouse and 17.1% of cases in Human (Table 2). In all other cases the match is not perfect. On average, a complementary match between the U1 nucleotides and the four variable nucleotides of the 5′ss is achieved in 67% of nucleotides. The highest match is with the second variable nucleotide (the fourth of the 5′ss) with 78% of occurrences, while the matching of all the other variable nucleotides averages 50%. Indeed, all seven more expressed 5′ss of Mouse and Human have an “A” in the fourth position (Table 2). The lowest matching is with the sixth nucleotide. However, the number of matches in the individual 5′sss is very variable, and in a few cases (e.g., GTTTCG) only the initial GT matches with U1. In very few cases we observed that the optimal ungapped base-pairing of U1 could be achieved by partly binding the last three nucleotides of the exon.

## 4. Discussion

This study was aimed at describing the conservation/mutation dynamics of the intron ends of the cytokine receptor genes during the speciation processes to Mouse and Human which began, starting from a common ancestor, 65–85 MYA [32, 33]. We selected 26 orthologous genes of the two species in which the Mouse/Human topographic correspondence of exons had been consistently preserved. The selected genes coded for receptors belonging to different cytokine receptor groups (Table 1). We firstly identified the nucleotide identities at the corresponding positions in Mouse and Human in the first 50 and last 50 nucleotides of each intron. All introns studied (216 couples) started and ended with the canonical dinucleotides GT and AG, respectively, conforming to the usual situation. This preliminary analysis demonstrated that, in general, the four nucleotides following GT at the 5′ss and the four nucleotides preceding AG at the 3′ss were more highly conserved, as compared to the other nucleotides at both ends of the introns (Figures 1-2). We thus undertook a more detailed analysis of the initial and terminal intronic hexanucleotides.

All the 16 hexanucleotides beginning by GT and ending by AG might act both as 5′ss and 3′ss. In our sample 8 of these sequences appeared either at 5′ or at 3′, but never in both. It may thus be hypothesized that some mechanism of disambiguation may operate possibly related to other exonic or intronic “signals” involved in the splicing processes.

The structure of the hexanucleotides actually expressed at the beginning and end of the introns is quite variable even if some configurations are more frequent (Tables 2 and 4). In our sample the initial hexanucleotide appeared in 51 different configurations while the terminal hexanucleotide appeared in 94 different configurations, indicating a lower selective pressure on the terminal hexanucleotide. This is confirmed by the Mouse/Human conservation/mutation data: the average mutation per hexanucleotide was 0.58 in the initial hexanucleotides and 1.17 in the terminal hexanucleotides, and the total hexanucleotide conservation was 57.4% for initial hexanucleotides and only 28.2% for terminal hexanucleotides.

The percentages of occurrence of the more expressed initial and terminal hexanucleotides did not differ significantly in Mouse and Human (Tables 2 and 4). The percentage of the individual nucleotides at each position in the initial and terminal hexanucleotides was also similar in Mouse and Human, and our results are in general comparable to those reported in the literature [25, 27, 28]. In our sample, in the sixth nucleotide of the initial hexanucleotides C was significantly more expressed in the Mouse than in Human, and in the first nucleotide of the terminal hexanucleotides G was significantly more expressed in the Mouse than in Human (Tables 3 and 5). In our sample the nucleotide G was never present in the fourth position of terminal hexanucleotides.

From the data on the actual frequencies of the individual nucleotides at each position in the initial and terminal hexanucleotides in Mouse and Human separately we calculated the probability of random conservation of any given nucleotide at a given position in both species. The results are shown in Table 3 (initial hexanucleotides) and Table 5 (terminal hexanucleotides), together with the results obtained on the actual conservation of each nucleotide at a given position in both Mouse and Human. As shown in the Tables the actual conservation was always significantly higher than the random conservation. On average, the actual conservation exceeded by 53% the random conservation in the initial hexanucleotides and by 80% in the terminal hexanucleotides. These “gains” express the conservation due to a selective evolutionary pressure to keep the configuration of the common ancestor at the level of the individual nucleotides.

We also analyzed the conservation of groups of the variable nucleotides in initial and terminal hexanucleotides. The random conservation of groups of three or two nucleotides was calculated and compared with the actual frequencies. All the motifs of the initial hexanucleotides found to be associated with a higher or a lower conservation are reported in the Results section. The most remarkably conserved trinucleotide was xxGAGx while xxAAGx was significantly underexpressed. Accordingly, GTGAGT and GTGAGG were overexpressed while GTAAGG was underexpressed. Since in the third position A is more conserved than G by about 70%, GTGAGT and GTGAGG should be less expressed than GTAAGT and GTAAGG, respectively, by a similar percentage. Actually GTGAGT and GTGAGG were more expressed than the similar sequences with A in the third position so that in our sample GTGAGT was the most frequently expressed initial hexanucleotide and GTAAGT was only the second most frequently occurring sequence (Table 2).

In the terminal hexanucleotides the sequences TTTxAG and xTGCAG were overexpressed, and accordingly TTTCAG, TTTTAG, and CTGCAG were more expressed than the random expectance. These three sequences are the most frequently expressed terminal hexanucleotides (Table 4). No specific motif entailing terminal nucleotide underexpression could be demonstrated.

It should be remarked that the presence of overexpressed or underexpressed trinucleotides seems to be a condition which, although necessary, is not sufficient per se to entail over- or underexpression of the corresponding hexanucleotides since the outcome also depends on the other variable nucleotide.

The present results suggest that the conservation of each nucleotide of the initial and terminal hexanucleotides is dependent upon the conservation/mutation of the other nucleotides, and an evolutionary selective pressure operates to keep certain global configurations derived from the Mouse/Human common ancestor while other configurations tend to be less conserved. In particular, the initial configuration GTAAGT, which corresponded to the maximal conservation of the individual nucleotides at positions 3 to 6 (Table 3, column 6) and is the one which exactly base-pairs with U1 snRNA, appeared to be negatively biased.

In general, the requirement for base-pairing of the initial hexanucleotide with U1 seems not to be stringent since in our sample complete matching was observed in 18% only of the 5′sss and 5′sss with as few as three matching nucleotides being effective (13% of cases). In very few cases, optimal base-pairing of U1 could be achieved by partly binding the terminal trinucleotide of the exon.

Analysis of the transcript variants of the genes considered in the present study revealed that no type of variant is common in Mouse and Human, except in one single case. This suggests that these variants were not inherited from the common ancestor but emerged only later during the speciation process. One frequent variant is the silencing of a constitutive 3′ss which leads to the skipping of the immediately downstream exon, with transcription being then resumed for the other downstream exons. Another type of variant is the activation of an alternative 5′ss or 3′ss, usually located a few nucleotides upstream or downstream of the canonical splicing site, the latter being silenced. This leads to alterations of the nucleotide number of the neighboring exons, but usually without frameshifts. Most constitutive sss, especially at 5′, are robust enough to be maintained steadily active, but 10.4% of the 3′sss and 3.2% of the 5′sss were found to be variable. Our analysis could not reveal any specific trend towards the silencing of constitutive sss or the activation of alternative sss, as certain hexanucleotides which are silenced at given sites are, on the contrary, activated at other sites to replace constitutive sss, as, for example, the 5′sss GTGAGA and GTGGGA and the 3′ss CTCCAG. Possibly, silencing or activation may depend in a complex way on the general context [20].

To conclude, analysis of the nucleotide conservation in the 5′ss and 3′ss hexanucleotides of Mouse and Human introns reveals definite evolutionary positive biases towards the conservation of some specific configurations and negative biases against other configurations. However, the 5′ss hexanucleotides and especially the 3′ss hexanucleotides still exhibit a wide structural variability, confirming the contention that splicing depends on a complex code which, besides the 5′ss and 3′ss, possibly involves additional signals from the neighboring exons or from sections of the intron other than the splice sites and might also be regulated by the secondary and tertiary structures forming in the pre-mRNA molecule [12–14, 16–23].

## Supplementary Material

Mouse and Human Transcript Variants of Cytokine Receptor Genes as compared to the Canonical.

## Figures and Tables

**Table 1:** The cytokine receptors analyzed in this study. (1) is the immunoglobulin superfamily receptors; (2) is the class I cytokine receptor family; (3) is the class II cytokine receptor family (interferon receptor family); (4) is the tumor necrosis factor receptor superfamily; (5) is the chemokine receptor family; (6) is the transforming growth factor beta receptor family.

RECEPTOR (Receptor family) (Number of exons)	
Interleukin 1 receptor, type I (IL1R1) (1) (10 exons)	
C-Kit Hardy-Zuckerman 4 Feline Sarcoma Viral Oncogene Homolog (KIT) (1) (21 exons)	
Interleukin 2 receptor, alpha (IL2RA) (2) (8 exons)	
Interleukin 2 receptor, beta (IL2RB) (2) (9 exons)	
Interleukin 2 receptor, gamma (IL2RG) (2) (8 exons)	
Interleukin 4 receptor (IL4R) (2) (9 exons)	
Interleukin 13 receptor, alpha 1 (IL13RA1) (2) (11 exons)	
Interleukin 13 receptor, alpha 2 (IL13RA2) (2) (9 exons)	
Erythropoietin receptor (EPOR) (2) (8 exons)	
Colony stimulating factor 2 receptor, beta, low-affinity (granulocyte-macrophage) (CSF2RB) (2) (13 exons)	
Colony stimulating factor 3 receptor (granulocyte) (CSF3R) (2) (15 exons)	
Leukemia inhibitory factor receptor alpha (LIFR) (2) (19 exons)	
Prolactin receptor (PRLR) (2) (8 exons)	
Oncostatin M receptor (OSMR) (2) (17 exons)	
Interleukin 10 receptor, alpha (IL10RA) (3) (7 exons)	
Interleukin 10 receptor, beta (IL10RB) (3) (7 exons)	
Interferon (alpha, beta, and omega) receptor 1 (IFNAR1) (3) (11 exons)	
CD27 molecule (CD27) (4) (6 exons)	
CD40 molecule, TNF receptor superfamily member 5 (CD40) (4) (9 exons)	
Lymphotoxin beta receptor (TNFR superfamily, member 3) (LTBR) (4) (10 exons)	
Chemokine (C-C motif) receptor 7 (CCR7) (5) (3 exons)	
Chemokine (C-C motif) receptor 9 (CCR9) (5) (2 exons)	
Chemokine (C-C motif) receptor 10 (CCR10) (5) (2 exons)	
Chemokine (C-X-C motif) receptor 4 (CXCR4) (5) (2 exons)	
Chemokine (C-X-C motif) receptor 5 (CXCR5) (5) (2 exons)	
Transforming growth factor, beta receptor III (TGFBR3) (6) (16 exons)	

**Table 2:** The initial more frequently expressed hexanucleotides. Percentages are over the total of 216 couples of hexanucleotides.

Sequence	% in Mouse	% in Human	Average (%)
GTGAGT	19.0	18.1	18.5
GTAAGT	19.0	17.1	18.1
GTAAGA	8.8	8.8	8.8
GTGAGA	6.0	5.1	5.6
GTGAGG	4.2	6.9	5.6
GTAAGG	3.7	5.1	4.4
GTAAGC	4.2	2.8	3.5
Total	**64.9**	**63.9**	**64.5**

**Table 3:** Columns 1–4: the nucleotide composition according to the position in the initial hexanucleotides in Mouse and Human. Columns 1 and 5–7: analysis of the conservation of individual nucleotides in the initial hexanucleotides of the introns. Expected random conservation in column 5 and actual conservation in column 6. Percentages are over the total of 216 couples of hexanucleotides.

1	2	3	4	5	6	7
Sequence	% in Mouse	% in Human		Random conservation (%)	Actual conservation (%)	
GTAxxx	59.7	57.5		34.03	50.93	*P* < 0.01
GTCxxx	1.9	2.3		0.04	1.85	*P* < 0.01
GTGxxx	37.0	38.4		14.21	30.09	*P* < 0.01
GTTxxx	1.4	2.3		0.03	1.39	*P* < 0.01
Total				48.31	84.26	*P* < 0.01

GTxAxx	82.4	81.0		66.74	77.78	*P* < 0.01
GTxCxx	2.8	2.8		0.08	2.31	*P* < 0.01
GTxGxx	6.9	8.3		0.57	4.17	*P* < 0.01
GTxTxx	7.9	7.9		0.62	6.02	*P* < 0.01
Total				68.01	90.28	*P* < 0.01

GTxxAx	7.9	7.4		0.58	6.48	*P* < 0.01
GTxxCx	1.4	1.8		0.03	0.93	*P* < 0.01
GTxxGx	85.2	84.3		71.82	82.41	*P* < 0.01
GTxxTx	5.5	6.5		0.36	3.70	*P* < 0.01
Total				72.79	93.52	*P* < 0.01

GTxxxA	21.8	24.1		5.25	16.20	*P* < 0.01
GTxxxC	11.6	7.4	*P* = 0.02	0.86	3.70	*P* < 0.01
GTxxxG	16.6	19.9		3.30	8.33	*P* < 0.01
GTxxxT	50.0	48.6		24.30	45.37	*P* < 0.01
Total				33.71	73.6	*P* < 0.01

**Table 4:** The terminal more frequently expressed hexanucleotides. Percentages are over the total of 216 couples of hexanucleotides.

Sequence	% in Mouse	% in Human	Average (%)
TTTCAG	7.4	6.5	6.9
CTGCAG	7.9	4.6	6.3
TTTTAG	5.1	6.0	5.6
TTGCAG	3.7	6.9	5.3
TTCTAG	3.2	6.5	4.9
CCACAG	4.6	3.2	3.9
TTCCAG	5.1	2.8	3.9
Total	**37.0**	**36.5**	**36.8**

**Table 5:** Columns 1–4: the nucleotide composition according to the position in the terminal hexanucleotides in Mouse and Human. Columns 1 and 5–7: analysis of the conservation of individual nucleotides in the terminal hexanucleotides of the introns. Expected random conservation in column 5 and actual conservation in column 6. Percentages are over the total of 216 couples of hexanucleotides.

1	2	3	4	5	6	7
Sequence	% in Mouse	% in Human		Random conservation (%)	Actual conservation (%)	
AxxxAG	5.1	5.6		0.29	2.31	*P* < 0.01
CxxxAG	35.2	33.8		11.9	23.61	*P* < 0.01
GxxxAG	8.3	4.6	*P* = 0.01	0.38	3.70	*P* < 0.01
TxxxAG	51.4	56.0		28.78	42.59	*P* < 0.01
Total				41.35	72.21	*P* < 0.01

xAxxAG	7.9	9.3		0.73	5.09	*P* < 0.01
xCxxAG	25.9	22.2		5.75	13.89	*P* < 0.01
xGxxAG	8.3	8.3		0.69	5.09	*P* < 0.01
xTxxAG	57.9	60.2		34.86	48.61	*P* < 0.01
Total				42.03	72.68	*P* < 0.01

xxAxAG	27.3	26.9		7.34	17.13	*P* < 0.01
xxCxAG	27.3	29.1		7.94	16.67	*P* < 0.01
xxGxAG	19.9	21.3		4.24	9.26	*P* < 0.01
xxTxAG	25.5	22.7		5.79	16.67	*P* < 0.01
Total				25.31	59.73	*P* < 0.01

xxxAAG	7.4	7.0		0.52	6.02	*P* < 0.01
xxxCAG	63.0	59.7		37.6	50.46	*P* < 0.01
xxxGAG	0.0	0.0		0.00	0.00	
xxxTAG	29.6	33.3		9.86	20.83	*P* < 0.01
Total				47.98	77.31	*P* < 0.01

**Table 6:** Conservation of groups of nucleotides (underlined) in the initial hexanucleotides of the introns. Percentages are over the total of 216 couples of hexanucleotides.

Sequence	Random probability (%)	Actual (%)	Actual-expected (%)	*P*
GTGAGx	19.29	25.46	6.17	0.02
GTGAxT	10.62	14.35	3.73	0.05
GTGxGT	11.25	15.28	4.03	0.04
GTAAGx	32.65	26.39	−6.26	0.03
GTAxGG	3.50	0.93	−2.57	0.02

**Table 7:** Conservation of groups of nucleotides (underlined) in the terminal hexanucleotides of the introns. Percentages are over the total of 216 couples of hexanucleotides.

Sequence	Random probability (%)	Actual (%)	Actual-expected (%)	*P*
TTTxAG	3.45 ± 2.68	8.80	5.35	<0.01
TTxTAG	4.31 ± 2.98	9.26	4.95	<0.01
xTTTAG	1.69 ± 1.89	4.63	2.94	<0.01
TxTTAG	1.48 ± 1.77	4.17	2.69	<0.01
xTGCAG	2.27 ± 2.19	5.09	2.82	0.01
TTxxAG	20.70 ± 5.95	29.63	8.93	<0.01
xxTTAG	3.47 ± 2.69	6.48	3.01	0.02
xTGxAG	4.50 ± 3.05	8.33	3.83	<0.01
