# Supplementary material for: Conservation/Mutation in the Splice Sites of Cytokine Receptor Genes of Mouse and Human
Source: Int J Evol Biol. 2013 Dec 17;2013:818954. doi: 10.1155/2013/818954 (PMC3877593; doi:10.1155/2013/818954)
Supplement: Supplementary file 1 — Mouse and Human Transcript Variants of Cytokine Receptor Genes as compared to the Canonical. [file 818954.f1.doc]

Supplementary Material

Mouse and Human Transcript Variants

**C-Kit Hardy-Zuckerman 4 Feline Sarcoma Viral Oncogene Homolog (KIT)**

In two Mouse and Human variants (Mouse transcript variant 2, NM_021099, and Human transcript variant X3, XM_005265742) the 5’ss after exon 9 is GTAACA while in the canonical forms (Mouse transcript variant 1, NM_001122733, and Human transcript variant 1, NM_000222) it is the GTATAT located 12 nt downstream.

**Interleukin 2 Receptor, Alpha (IL2RA)**

Human transcript variant X1 (XM_005252446) lacks exon 4 of the canonical sequence (NM_000417) as the 3’ss TTCCAG immediately upstream of this exon (216 nt; 72 aa) is silenced.

Human transcript variant X2 (XM_005252447) lacks exons 4 and 5 of the canonical sequence (NM_000417) as the 3’sss TTCCAG and CCCCAG immediately upstream of these exons (216 nt and 72 nt, respectively; 96 aa) are both silenced.

**Interleukin 2 Receptor, Gamma (IL2RG)**

In Human transcript variant X1 (XM_005262261) after the first exon the 5’ss is GTGAGA while in the canonical form (NM_000206) it is the GTGGGA located 28 nt upstream. The length of the first exon canonical form is 115 nt (38.33 aa); in this variant transcription starts at nucleotide 92 of the canonical sequence and thus the first exon total length is 115 – 91 + 28 = 52 nt (17.33 aa); thus canonical and variant transcripts continue in-frame after the first exon.

Human transcript variant X2 (XM_005262262) lacks exons 2, 3, and 4 of the canonical sequence (NM_000206) as the 3’sss ATCTAG, CTCTAG and CTCCAG immediately upstream of these exons (154, 185, and 140 nt, respectively; 159.67 aa) are all silenced. Transcription of the variant starts at nucleotide 92 of the canonical sequence. The sum of nucleotides in the first four exons of the canonical sequence is 594 (198 aa) and the length of the first exon of the variant is 115 - 91 = 24 nt (8 aa). The two sequences are thus in frame starting from the fifth exon of the canonical and the second exon of the variant.

**Interleukin 4 Receptor (IL4R)**

In Human transcript variant X3 (XM_005255307) transcription starts at an internal ATG of the second exon of the canonical form (transcript variant 1, NM_000418). In this variant exon 4 of the canonical form is skipped as the 3’ss CCGCAG immediately upstream of this exon is silenced. In the variant the total number of nucleotides in exons 1 and 2 is 216 (72 aa); in the canonical form the total number of nucleotides in exons 1-4 is 513 (171 aa). Then transcription continues in-frame in all other exons.

In Human transcript variant X5 (XM_005255309) coding stars in the 5’ UTR of the canonical form (transcript variant 1, NM_000418) and continues uninterruptedly and in-frame in the exon 1-7 of the canonical sequence. Then exon 8 (50 nt, 16.67 aa) is skipped as the 3’ss TTTTAG immediately upstream of this exon is silenced, resulting in a frameshift in exon 9 and termination due to a stop codon.

**Colony Stimulating Factor 2 Receptor, Beta, Low-Affinity (Granulocyte-Macrophage) (CSF2RB)**

In Human variant X1 (XM_005261340) the intron upstream of exon 7 ends by an “early” CCTCAG while the corresponding intron in the canonical sequence (NM_007780) ends at another CCTCAG 18 nt downstream.

Human transcript variant X2 (XM_005261341) lacks the two first exons and starts with a short sequence present in the intron located between exons 2 and 3 of the canonical sequence NM_007780. This initial coding sequence (23 nt, 7.67 aa; followed by a 5’ss GTGAGG) continues in frame with the canonical exon 3 (since canonical exons 1+2 = 200 nt, 66.67 aa).

**Colony Stimulating Factor 3 Receptor (Granulocyte) (CSF3R)**

Mouse transcript variant 2 (NM_001252651) lacks exons 5 and 6 of the canonical sequence (NM_007782) as the 3’ss CTGCAG and CACCAG immediately upstream of these exons (170 nt and 154 nt, respectively; 108 aa) are both silenced.

In Human transcript variant X1 (XM_005270492) the 3’ss CCCCAG of the first intron of the canonical form (transcript variant 1, NM_000760) is silenced and first intron stops further downstream at an TCACAG which is within the second canonical exon, with a loss of 66 nt. Furthermore, this variant also exhibits an additional 81 nt insert as in variants 3 and X4 (see below).

In Human transcript variants 3 (NM_156039) and X4 (XM_005270495) the last intron stops at an internal CCACAG, 81 nt upstream of the 3’ss GTGCAG of the canonical form **(**transcript variant 1, NM_000760); then coding continues uninterruptedly as in the canonical form.

In Human transcript variant 4 (NM_172313) the last exon of the canonical sequence **(**transcript variant 1, NM_000760) is stopped at an internal GTCCTT, but this transcript exhibits an additional exon between a CCATAG and the stop codon TAA, in a region corresponding to the canonical 3’ UTR.

**Leukemia Inhibitory Factor Receptor Alpha (LIFR)**

In Mouse transcript variant 2 (NM_001113386) the intron following the exon 14 of the canonical sequence (transcript variant 1, NM_013584) ends at an internal TTACAG and transcription continues up to an internal stop codon; as a consequence, all the other exons (15 to 19) are lacking.

**Prolactin Receptor (PRLR)**

In Mouse transcript variant 2 (NM_001253781) at the end of intron 7 the 3’ss of the canonical sequence CAATAG (transcript variant 1, NM_011169) is silenced. Thus canonical exon 8 is not transcribed but the variant coding sequence resumes 19,864 nt downstream after AATTAG till a stop codon is reached.

In Mouse transcript variant 3 (NM_001253782) at the end of intron 7 the 3’ss of the canonical sequence CAATAG (transcript variant 1, NM_011169) is silenced. Thus canonical exon 8 is not transcribed but the variant coding sequence resumes 19,114 nt downstream after TTTCAG till a stop codon is reached.

In Human transcript variant 2 (NM_001204315) the last exon stops at an internal GTATGA of the canonical sequence (transcript variant 1, NM_000949); then transcription resumes for an additional exon after an AAACAG in the 3’ UTR region of the canonical sequence.

In Human transcript variant 3 (NM_001204316) the last exon stops at an internal GTATGA of the canonical sequence (transcript variant 1, NM_000949); then transcription resumes for an additional exon after a CTCCAG in the 3’ UTR region of the canonical sequence.

In Human transcript variant 4 (NM_001204317) the last exon of the canonical sequence (transcript variant 1, NM_000949) is missing as the upstream 3’ss TAATAG is silenced; however, transcription resumes after a CTCCAG in the 3’ UTR region of the canonical sequence.

In Human transcript variant 5 (NM_001204318) exons 6, 7 and 8 of the canonical sequence (transcript variant 1, NM_000949) are missing as the corresponding 3’sss TTTTAG, TTATAG and TAATAG are all silenced; however, transcription resumes for a further exon after a CTCCAG in the 3’ UTR region of the canonical sequence.

Human transcript variant 6 (NM_001204314) lacks exons 2 and 3 of the canonical sequence (transcript variant 1, NM_000949) as the 3’sss TTCTAG and TTGCAG immediately upstream of these exons (133 nt and 170 nt, respectively; 101 aa) are both silenced.

**Oncostatin M Receptor (OSMR)**

In Human transcript variant X3 (XM_005248386) the intron following the exon 7 ends by ATCAAG, three positions upstream of the AAGCAG of the canonical form (transcript variant 1, NM_003999); thus exon 8 has three more nt at the beginning as compared to the canonical sequence. Furthermore, like the variant X4 (see below), all exons of the canonical form after the tenth are missing and an additional exon is transcribed after a GGCCAG from a section of the intron following exon 10 of the canonical sequence.

In Human transcript variant X4 (XM_005248387) in the intron following exon 10 of the canonical form (transcript variant 1, NM_003999) a novel 3’ss GGCCAG is activated and a part of the intron is transcribed till a stop codon is reached. Thus all exons after the tenth (11-17) are missing.

In Human transcript variant 2 (NM_001168355) the 5’ss GTGAGA after exon 6 of the canonical form (transcript variant 1, NM_003999) is silenced and the exon continues up to a stop codon; canonical exons 7-17 are thus missing.

**CD40 Molecule, TNF Receptor Superfamily Member 5 (CD40)**

Mouse transcript variant 2 (NM_170703) lacks exon 6 of the canonical sequence (transcript variant 1, NM_011611) as the 3’ss GTGCAG immediately upstream of this exon is silenced. This determines a frameshift and an in-frame stop codon is reached in exon 8. Thus exon 9 is lacking.

In Mouse transcript variant 4 (NM_170704) after the exon 7 the 3’ss GAAAAG replaces the 3’ss CACTAG of the canonical variant (transcript variant 1, NM_011611) which occurs five nt upstream. This determines a frameshift and an in-frame stop codon in exon 8. Thus exon 9 is lacking.

Mouse transcript variant 5 (NM_170702) lacks exon 7 of the canonical sequence (transcript variant 1, NM_011611) as the 3’ss CTCCAG immediately upstream of this exon (87 nt) is silenced. Transcription resumes in-frame in exons 8 and 9.

In Human transcript variant X1 (XM_005260617) the 5’ss after exon 7 is GTGGGG, replacing the GTGAGT of the canonical variant (transcript variant 1, NM_001250) which occurs 12 nt upstream.

In Human transcript variant X2 (XM_005260618) the canonical 5’ss GTGAGT after exon 6 (transcript variant 1, NM_001250) is replaced by GTGGGA which is located 40 nt downstream. The ensuing frameshift leads to a stop codon in exon 8.

Human transcript variant X3 (XM_005260619) lacks exons 5 and 6 of the canonical variant (transcript variant 1, NM_001250) as the 3’sss TCCCAG and GCATAG, which are immediately upstream of exons 5 and 6 (156 nt, 52 aa), are silenced. Furthermore, like in variant X1 (see above), the 5’ss after the seventh exon is GTGGGG, replacing the GTGAGT of the canonical variant which occurs 12 nt upstream.

In Human transcript variant X4 (XM_005260620) the canonical 3’ss TCCCAG upstream of exon 5 (transcript variant 1, NM_001250) is silenced. In addition, the canonical 5’ss GTGAGT downstream of exon 6 is also silenced. The loss of exon 5 (94 nt) determines a frameshift which finally leads to a stop codon in the prolonged exon 6.

In Human transcript variant 2 (NM_152854) the canonical 3’ss GCATAG upstream of exon 6 (transcript variant 1, NM_001250) is silenced. The loss of exon 6 (62 nt) determines a frameshift which leads to a stop codon in exon 8.

**Lymphotoxin Beta Receptor (TNFR Superfamily, Member 3) (LTBR)**

In Human transcript variant X1 (XM_005253687) the canonical 3’ss TTCTAG upstream of exon 3 (NM_002342) is replaced by TCTCAG which is located 15 nt downstream.

Human transcript variant X2 (XM_005253688) lacks exon 7 (108 nt, 36 aa) of the canonical sequence (NM_002342) as the 3’ss TGGCAG immediately upstream of this exon is silenced.

**Transforming Growth Factor, Beta Receptor III (TGFBR3)**

In Human transcript variant 2 (NM_001195683) the canonical 3’ss CACCAG upstream of exon 8 (transcript variant 1, NM_003243) is replaced by CAGCAG which is located 3 nt downstream.
